# Supplementary material for: Prevalence of Functional Difficulty Among School-Aged Children and Effect on School Enrolment in Rural Southern India: A Cross-Sectional Analysis
Source: J Epidemiol Glob Health. 2024 Sep 19;14(4):1476–89. doi: 10.1007/s44197-024-00293-7 (PMC11652546; doi:10.1007/s44197-024-00293-7)
Supplement: Supplementary file 1 — Supplementary Material 1 [file 44197_2024_293_MOESM1_ESM.docx]

Table A1: Modification of the effect of functional-difficulty domain 'Seeing' on school non-enrolment by selected sociodemographic factors among N=29,044 children in Tamil Nadu, India

(children with other functional limitations have been excluded).

|  | **No  Functional Difficulty** | | **Any  Functional Difficulty** | | **Effect of FD** | **EM Measures** | |
| --- | --- | --- | --- | --- | --- | --- | --- |
|  | **Non-enrolment  n/N**  **(%)** | **PR**  **(95% CI)** | **Non-enrolment  n/N**  **(%)** | **PR**  **(95% CI)** | **PR**  **(95% CI)** | **Multiplicative** | **Additive** |
| Overall (crude) | 2,731/28,745  (10) | 1 | 9/34  (26) | 2.79  (1.59, 4.88) |  |  |  |
| Overall (adjusted)* | 2,731/28,745  (10) | 1 | 9/34  (26) | 3.16  (1.89, 5.29) |  |  |  |
| **Age Category**  **(years)** |  |  |  |  |  | 0.13  (0.03, 0.62) | -7.27  (-27.07, 11.68) |
| 5-11 | 328/12,742  (2.6) | 1 | 5/18  (27.8) | 10.30  (3.62, 29.29) | 10.30  (3.62, 29.29) |  |  |
| 12-17 | 2,523/16,268  (16) | 6.04  (5.27, 6.92) | 4/16  (25) | 8.07  (2.51, 25.92) | 1.34  (0.42, 4.27) |  |  |
| **Gender** |  |  |  |  |  | 0.69  (0.15, 3.29) | -0.70  (-6.62, 5.34) |
| Female | 1,206/13,953  (8.6) | 1 | 5/18  (27.8) | 3.10  (1.10, 8.74) | 3.10  (1.10, 8.74) |  |  |
| Male | 1,645/15,057  (11) | 1.21  (1.11, 1.33) | 4/16  (25) | 2.61  (0.82, 8.32) | 2.15  (0.67, 6.85) |  |  |
| **Socioeconomic Status** |  |  |  |  |  | 2.12  (0.18, 24.28) | 3.32  (-9.09, 10.57) |
| Less poor  (Upper 2 quintiles) | 919/17,547  (5.2) | 1 | 1/16  (6.2) | 1.34  (0.13, 13.35) | 1.34  (0.13, 13.35) |  |  |
| Poorer  (Lower 3 quintiles) | 1,932/11,463  (17) | 1.99  (1.76, 2.25) | 8/18  (44) | 5.65  (2.49, 12.82) | 2.84  (1.26, 6.41) |  |  |
| **No HoH Education** |  |  |  |  |  | 0.38  (0.08, 1.94) | -1.84  (-8.52, 7.66) |
| Any education | 1,390/22,127  (6.3) | 1 | 6/24  (25.0) | 4.00  (1.57, 10.21) | 4.00  (1.57, 10.21) |  |  |
| No education | 1,461/6,883  (21) | 2.17  (1.97, 2.40) | 3/10  (30) | 3.34  (0.89, 12.53) | 1.53  (0.41, 5.76) |  |  |
| **Site** |  |  |  |  |  | 1.51  (0.23, 9.68) | 5.29  (-4.38, 18.72) |
| Timiri | 1,230/20,729  (5.9) | 1 | 2/21  (9.5) | 1.91  (0.37, 9.84) | 1.91  (0.37, 9.84) |  |  |
| Jawadhu Hills | 1,621/8,281  (20) | 3.30  (3.02, 3.60) | 7/13  (54) | 9.50  (3.95, 22.86) | 2.88  (1.20, 6.93) |  |  |

*Adjusted for age category, gender, and site; PR = Prevalence Ratio; CI = Confidence Interval; FD = Functional Difficulty; EM = Effect Modification

Table A2: Modification of the effect of functional-difficulty domain 'Hearing' on school non-enrolment by selected sociodemographic factors among N=29,044 children in Tamil Nadu, India

(children with other functional limitations have been excluded).

|  | **No  Functional Difficulty** | | **Any  Functional Difficulty** | | **Effect of FD** | | | **EM Measures** | |
| --- | --- | --- | --- | --- | --- | --- | --- | --- | --- |
|  | **Non-enrolment  n/N**  **(%)** | **PR**  **(95% CI)** | **Non-enrolment  n/N**  **(%)** | **PR**  **(95% CI)** | **PR**  **(95% CI)** | **Multiplicative** | **Additive** | |  |
| Overall  (crude) | 2,731/28,745  (10) | 1 | 18/43  (42) | 4.41  (3.08, 6.30) |  |  |  | |  |
| Overall  (adjusted)* | 2,731/28,745  (10) | 1 | 18/43  (42) | 4.41  (2.85, 6.82) |  |  |  | |  |
| **Age Category**  **(years)** |  |  |  |  |  | 0.28  (0.09, 0.89) | 2.06  (-16.76, 20.25) | |  |
| 5-11 | 327/12,743  (2.6) | 1 | 6/17  (35.3) | 10.55  (4.06, 27.41) | 10.55  (4.06, 27.41) |  |  | |  |
| 12-17 | 2,515/16,258  (15) | 6.04  (5.27, 6.92) | 12/26  (46) | 17.65  (8.93, 34.90) | 2.92  (1.49, 5.72) |  |  | |  |
| **Gender** |  |  |  |  |  | 1.29  (0.40, 4.12) | 1.82  (-3.00, 10.81) | |  |
| Female | 1,199/13,944  (8.6) | 1 | 12/27  (44.4) | 3.56  (1.82, 6.97) | 3.56  (1.82, 6.97) |  |  | |  |
| Male | 1,643/15,057  (11) | 1.22  (1.12, 1.33) | 6/16  (38) | 5.60  (2.17, 14.44) | 4.60  (1.78, 11.85) |  |  | |  |
| **Socioeconomic Status** |  |  |  |  |  | 0.67  (0.22, 1.99) | 0.73  (-6.73, 8.76) | |  |
| Less poor  (Upper 2 quintiles) | 912/17,544  (5.2) | 1 | 8/19  (42.1) | 5.21  (2.30, 11.78) | 5.21  (2.30, 11.78) |  |  | |  |
| Poorer  (Lower 3 quintiles) | 1,930/11,457  (17) | 2.00  (1.77, 2.27) | 10/24  (42) | 6.95  (3.33, 14.49) | 3.47  (1.67, 7.18) |  |  | |  |
| **No HoH Education** |  |  |  |  |  | 0.39  (0.12, 1.24) | -2.01  (-8.21, 6.14) | |  |
| Any education | 1,384/22,120  (6.3) | 1 | 12/31  (38.7) | 5.77  (2.97, 11.20) | 5.77  (2.97, 11.20) |  |  | |  |
| No education | 1,458/6,881  (21) | 2.18  (1.98, 2.40) | 6/12  (50) | 4.94  (1.93, 12.62) | 2.27  (0.89, 5.78) |  |  | |  |
| **Site** |  |  |  |  |  | 0.45  (0.15, 1.34) | 0.55  (-7.59, 12.20) | |  |
| Timiri | 1,222/20,725  (5.9) | 1 | 10/25  (40.0) | 5.97  (2.86, 12.46) | 5.97  (2.86, 12.46) |  |  | |  |
| Jawadhu Hills | 1,620/8,276  (20) | 3.32  (3.04, 3.62) | 8/18  (44) | 8.84  (3.89, 20.10) | 2.67  (1.17, 6.06) |  |  | |  |

*Adjusted for age category, gender, and site; PR = Prevalence Ratio; CI = Confidence Interval; FD = Functional Difficulty; EM = Effect Modification

Table A3: Modification of the effect of functional-difficulty domain 'Walking' on school non-enrolment by selected sociodemographic factors among N=29,044 children in Tamil Nadu, India

(children with other functional limitations have been excluded).

|  | **No  Functional Difficulty** | | **Any  Functional Difficulty** | | **Effect of FD** | **EM Measures** | |
| --- | --- | --- | --- | --- | --- | --- | --- |
|  | **Non-enrolment  n/N**  **(%)** | **PR**  **(95% CI)** | **Non-enrolment  n/N**  **(%)** | **PR**  **(95% CI)** | **PR**  **(95% CI)** | **Multiplicative** | **Additive** |
| Overall  (crude) | 2,731/28,745  (10) | 1 | 69/110  (63) | 6.60  (5.67, 7.68) |  |  |  |
| Overall  (adjusted)* | 2,731/28,745  (10) | 1 | 69/110  (63) | 7.66  (6.03, 9.72) |  |  |  |
| **Age Category**  **(years)** |  |  |  |  |  | 0.28  (0.15, 0.51) | 8.36  (-5.88, 23.30) |
| 5-11 | 309/12,713  (2.4) | 1 | 24/47  (51.1) | 18.10  (11.12, 29.45) | 18.10  (11.12, 29.45) |  |  |
| 12-17 | 2,482/16,221  (15) | 6.30  (5.49, 7.24) | 45/63  (71) | 31.76  (22.02, 45.82) | 5.04  (3.57, 7.12) |  |  |
| **Gender** |  |  |  |  |  | 0.91  (0.52, 1.59) | 0.55  (-3.65, 4.95) |
| Female | 1,176/13,916  (8.5) | 1 | 35/55  (63.6) | 7.06  (4.77, 10.44) | 7.06  (4.77, 10.44) |  |  |
| Male | 1,615/15,018  (11) | 1.22  (1.12, 1.33) | 34/55  (62) | 7.83  (5.26, 11.66) | 6.41  (4.31, 9.53) |  |  |
| **Socioeconomic Status** |  |  |  |  |  | 0.43  (0.25, 0.76) | -2.27  (-8.05, 3.60) |
| Less poor  (Upper 2 quintiles) | 882/17,502  (5.0) | 1 | 38/61  (62.3) | 10.71  (7.35, 15.60) | 10.71  (7.35, 15.60) |  |  |
| Poorer  (Lower 3 quintiles) | 1,909/11,432  (17) | 2.04  (1.81, 2.31) | 31/49  (63) | 9.48  (6.22, 14.46) | 4.64  (3.08, 7.00) |  |  |
| **No HoH Education** |  |  |  |  |  | 0.28  (0.15, 0.50) | -5.49  (-10.48, -0.16) |
| Any education | 1,347/22,073  (6.1) | 1 | 49/78  (62.8) | 10.95  (7.88, 15.21) | 10.95  (7.88, 15.21) |  |  |
| No education | 1,444/6,861  (21) | 2.21  (2.01, 2.44) | 20/32  (62) | 6.67  (4.00, 11.10) | 3.02  (1.81, 5.02) |  |  |
| **Site** |  |  |  |  |  | 0.38  (0.21, 0.70) | 0.59  (-5.88, 9.51) |
| Timiri | 1,184/20,675  (5.7) | 1 | 48/75  (64.0) | 10.03  (7.16, 14.05) | 10.03  (7.16, 14.05) |  |  |
| Jawadhu Hills | 1,607/8,259  (19) | 3.39  (3.11, 3.70) | 21/35  (60) | 13.01  (7.86, 21.54) | 3.84  (2.32, 6.34) |  |  |

*Adjusted for age category, gender, and site; PR = Prevalence Ratio; CI = Confidence Interval; FD = Functional Difficulty; EM = Effect Modification

Table A4: Modification of the effect of functional-difficulty domain 'Self-Care' on school non-enrolment by selected sociodemographic factors among N=29,044 children in Tamil Nadu, India

(children with other functional limitations have been excluded).

|  | **No  Functional Difficulty** | | **Any  Functional Difficulty** | | **Effect of FD** | **EM Measures** | |
| --- | --- | --- | --- | --- | --- | --- | --- |
|  | **Non-enrolment  n/N**  **(%)** | **PR**  **(95% CI)** | **Non-enrolment  n/N**  **(%)** | **PR**  **(95% CI)** | **PR**  **(95% CI)** | **Multiplicative** | **Additive** |
| Overall  (crude) | 2,731/28,745  (10) | 1 | 73/99  (74) | 7.76  (6.83, 8.82) |  |  |  |
| Overall  (adjusted)* | 2,731/28,745  (10) | 1 | 73/99  (74) | 10.99  (9.07, 13.32) |  |  |  |
| **Age Category**  **(years)** |  |  |  |  |  | 0.28  (0.16, 0.49) | 14.94  (-4.07, 36.34) |
| 5-11 | 304/12,712  (2.4) | 1 | 29/48  (60.4) | 25.51  (16.36, 39.79) | 25.51  (16.36, 39.79) |  |  |
| 12-17 | 2,483/16,233  (15) | 6.40  (5.57, 7.36) | 44/51  (86) | 45.86  (31.70, 66.35) | 7.16  (5.05, 10.14) |  |  |
| **Gender** |  |  |  |  |  | 1.10  (0.64, 1.90) | 3.01  (-3.04, 9.31) |
| Female | 1,179/13,927  (8.5) | 1 | 32/44  (72.7) | 9.48  (6.30, 14.24) | 9.48  (6.30, 14.24) |  |  |
| Male | 1,608/15,018  (11) | 1.21  (1.11, 1.32) | 41/55  (75) | 12.70  (8.84, 18.24) | 10.46  (7.29, 15.01) |  |  |
| **Socioeconomic Status** |  |  |  |  |  | 0.42  (0.24, 0.73) | -3.13  (-10.64, 4.86) |
| Less poor  (Upper 2 quintiles) | 876/17,504  (5.0) | 1 | 44/59  (74.6) | 14.91  (10.53, 21.12) | 14.91  (10.53, 21.12) |  |  |
| Poorer  (Lower 3 quintiles) | 1,911/11,441  (17) | 2.03  (1.80, 2.30) | 29/40  (72) | 12.82  (8.35, 19.68) | 6.31  (4.13, 9.62) |  |  |
| **No HoH Education** |  |  |  |  |  | 0.34  (0.18, 0.64) | -4.74  (-11.37, 3.92) |
| Any education | 1,339/22,078  (6.1) | 1 | 57/73  (78.1) | 13.97  (10.31, 18.93) | 13.97  (10.31, 18.93) |  |  |
| No education | 1,448/6,867  (21) | 2.22  (2.01, 2.44) | 16/26  (62) | 10.45  (5.94, 18.38) | 4.72  (2.68, 8.31) |  |  |
| **Site** |  |  |  |  |  | 0.46  (0.24, 0.89) | 5.01  (-4.74, 20.76) |
| Timiri | 1,175/20,672  (5.7) | 1 | 57/78  (73.1) | 12.57  (9.23, 17.11) | 12.57  (9.23, 17.11) |  |  |
| Jawadhu Hills | 1,612/8,273  (19) | 3.42  (3.14, 3.74) | 16/21  (76) | 20.00  (11.27, 35.48) | 5.84  (3.30, 10.35) |  |  |

*Adjusted for age category, gender, and site; PR = Prevalence Ratio; CI = Confidence Interval; FD = Functional Difficulty; EM = Effect Modification

Table A5: Modification of the effect of functional-difficulty domain 'Being Understood' on school non-enrolment by selected sociodemographic factors among N=29,044 children in Tamil Nadu, India

(children with other functional limitations have been excluded).

|  | **No  Functional Difficulty** | | **Any  Functional Difficulty** | | **Effect of FD** | **EM Measures** | |
| --- | --- | --- | --- | --- | --- | --- | --- |
|  | **Non-enrolment  n/N**  **(%)** | **PR**  **(95% CI)** | **Non-enrolment  n/N**  **(%)** | **PR**  **(95% CI)** | **PR**  **(95% CI)** | **Multiplicative** | **Additive** |
| Overall  (crude) | 2,731/28,745  (10) | 1 | 77/129  (60) | 6.28  (5.42, 7.29) |  |  |  |
| Overall  (adjusted)* | 2,731/28,745  (10) | 1 | 77/129  (60) | 6.51  (5.10, 8.31) |  |  |  |
| **Age Category**  **(years)** |  |  |  |  |  | 0.21  (0.12, 0.37) | 2.23  (-10.94, 15.39) |
| 5-11 | 303/12,701  (2.4) | 1 | 30/59  (50.8) | 20.16  (13.02, 31.23) | 20.16  (13.02, 31.23) |  |  |
| 12-17 | 2,480/16,214  (15) | 6.42  (5.59, 7.38) | 47/70  (67) | 27.82  (19.44, 39.81) | 4.33  (3.09, 6.06) |  |  |
| **Gender** |  |  |  |  |  | 1.00  (0.59, 1.70) | 1.16  (-2.51, 4.95) |
| Female | 1,175/13,910  (8.4) | 1 | 36/61  (59.0) | 6.23  (4.24, 9.15) | 6.23  (4.24, 9.15) |  |  |
| Male | 1,608/15,005  (11) | 1.22  (1.12, 1.33) | 41/68  (60) | 7.60  (5.30, 10.90) | 6.24  (4.36, 8.94) |  |  |
| **Socioeconomic Status** |  |  |  |  |  | 0.46  (0.27, 0.78) | -1.59  (-6.85, 3.54) |
| Less poor  (Upper 2 quintiles) | 882/17,493  (5.0) | 1 | 38/70  (54.3) | 9.93  (6.83, 14.43) | 9.93  (6.83, 14.43) |  |  |
| Poorer  (Lower 3 quintiles) | 1,901/11,422  (17) | 2.04  (1.81, 2.31) | 39/59  (66) | 9.38  (6.44, 13.65) | 4.59  (3.19, 6.61) |  |  |
| **No HoH Education** |  |  |  |  |  | 0.24  (0.13, 0.43) | -6.25  (-10.72, -1.61) |
| Any education | 1,340/22,061  (6.1) | 1 | 56/90  (62.2) | 10.84  (7.98, 14.72) | 10.84  (7.98, 14.72) |  |  |
| No education | 1,443/6,854  (21) | 2.23  (2.02, 2.45) | 21/39  (54) | 5.81  (3.54, 9.54) | 2.61  (1.59, 4.27) |  |  |
| **Site** |  |  |  |  |  | 0.31  (0.18, 0.55) | -1.73  (-7.42, 5.47) |
| Timiri | 1,179/20,662  (5.7) | 1 | 53/88  (60.2) | 10.50  (7.63, 14.45) | 10.50  (7.63, 14.45) |  |  |
| Jawadhu Hills | 1,604/8,253  (19) | 3.40  (3.12, 3.71) | 24/41  (59) | 11.17  (6.99, 17.86) | 3.28  (2.06, 5.24) |  |  |

*Adjusted for age category, gender, and site; PR = Prevalence Ratio; CI = Confidence Interval; FD = Functional Difficulty; EM = Effect Modification

Table A6: Modification of the effect of functional-difficulty domain 'Learning' on school non-enrolment by selected sociodemographic factors among N=29,044 children in Tamil Nadu, India

(children with other functional limitations have been excluded).

|  | **No  Functional Difficulty** | | **Any  Functional Difficulty** | | **Effect of FD** | **EM Measures** | |
| --- | --- | --- | --- | --- | --- | --- | --- |
|  | **Non-enrolment  n/N**  **(%)** | **PR**  **(95% CI)** | **Non-enrolment  n/N**  **(%)** | **PR**  **(95% CI)** | **PR**  **(95% CI)** | **Multiplicative** | **Additive** |
| Overall  (crude) | 2,731/28,745  (10) | 1 | 66/100  (66) | 6.95  (5.99, 8.06) |  |  |  |
| Overall  (adjusted)* | 2,731/28,745  (10) | 1 | 66/100  (66) | 8.79  (6.90, 11.19) |  |  |  |
| **Age Category**  **(years)** |  |  |  |  |  | 0.24  (0.13, 0.44) | 7.95  (-10.48, 26.99) |
| 5-11 | 308/12,713  (2.4) | 1 | 25/47  (53.2) | 24.77  (15.39, 39.86) | 24.77  (15.39, 39.86) |  |  |
| 12-17 | 2,486/16,231  (15) | 6.33  (5.52, 7.27) | 41/53  (77) | 38.05  (26.00, 55.68) | 6.01  (4.19, 8.62) |  |  |
| **Gender** |  |  |  |  |  | 1.32  (0.74, 2.32) | 4.21  (-1.08, 10.41) |
| Female | 1,179/13,922  (8.5) | 1 | 32/49  (65.3) | 7.38  (4.91, 11.09) | 7.38  (4.91, 11.09) |  |  |
| Male | 1,615/15,022  (11) | 1.22  (1.12, 1.33) | 34/51  (67) | 11.81  (7.94, 17.56) | 9.70  (6.53, 14.41) |  |  |
| **Socioeconomic Status** |  |  |  |  |  | 0.50  (0.29, 0.89) | -0.72  (-7.52, 6.80) |
| Less poor  (Upper 2 quintiles) | 882/17,501  (5.0) | 1 | 38/62  (61.3) | 11.92  (8.20, 17.33) | 11.92  (8.20, 17.33) |  |  |
| Poorer  (Lower 3 quintiles) | 1,912/11,443  (17) | 2.03  (1.80, 2.30) | 28/38  (74) | 12.24  (7.91, 18.94) | 6.02  (3.91, 9.26) |  |  |
| **No HoH Education** |  |  |  |  |  | 0.31  (0.16, 0.62) | -4.95  (-10.88, 2.77) |
| Any education | 1,344/22,075  (6.1) | 1 | 52/76  (68.4) | 12.20  (8.87, 16.77) | 12.20  (8.87, 16.77) |  |  |
| No education | 1,450/6,869  (21) | 2.22  (2.01, 2.44) | 14/24  (58) | 8.46  (4.62, 15.50) | 3.82  (2.09, 6.99) |  |  |
| **Site** |  |  |  |  |  | 0.36  (0.18, 0.71) | 0.07  (-7.74, 12.41) |
| Timiri | 1,180/20,670  (5.7) | 1 | 52/80  (65.0) | 11.69  (8.46, 16.14) | 11.69  (8.46, 16.14) |  |  |
| Jawadhu Hills | 1,614/8,274  (20) | 3.41  (3.13, 3.73) | 14/20  (70) | 14.17  (7.67, 26.16) | 4.15  (2.25, 7.65) |  |  |

*Adjusted for age category, gender, and site; PR = Prevalence Ratio; CI = Confidence Interval; FD = Functional Difficulty; EM = Effect Modification

Table A7: Modification of the effect of functional-difficulty domain 'Remembering' on school non-enrolment by selected sociodemographic factors among N=29,044 children in Tamil Nadu, India

(children with other functional limitations have been excluded).

|  | **No  Functional Difficulty** | | **Any  Functional Difficulty** | | **Effect of FD** | **EM Measures** | |
| --- | --- | --- | --- | --- | --- | --- | --- |
|  | **Non-enrolment  n/N**  **(%)** | **PR**  **(95% CI)** | **Non-enrolment  n/N**  **(%)** | **PR**  **(95% CI)** | **PR**  **(95% CI)** | **Multiplicative** | **Additive** |
| Overall  (crude) | 2,731/28,745  (10) | 1 | 68/92  (74) | 7.78  (6.80, 8.91) |  |  |  |
| Overall  (adjusted)* | 2,731/28,745  (10) | 1 | 68/92  (74) | 8.94  (6.99, 11.43) |  |  |  |
| **Age Category**  **(years)** |  |  |  |  |  | 0.18  (0.10, 0.32) | -1.62  (-22.96, 17.32) |
| 5-11 | 308/12,721  (2.4) | 1 | 25/39  (64.1) | 31.68  (19.69, 50.97) | 31.68  (19.69, 50.97) |  |  |
| 12-17 | 2,484/16,231  (15) | 6.34  (5.52, 7.27) | 43/53  (81) | 35.39  (24.39, 51.35) | 5.59  (3.93, 7.94) |  |  |
| **Gender** |  |  |  |  |  | 1.38  (0.79, 2.42) | 4.42  (-0.53, 10.12) |
| Female | 1,180/13,927  (8.5) | 1 | 31/44  (70.5) | 6.81  (4.50, 10.29) | 6.81  (4.50, 10.29) |  |  |
| Male | 1,612/15,025  (11) | 1.21  (1.11, 1.32) | 37/48  (77) | 11.44  (7.83, 16.72) | 9.42  (6.45, 13.75) |  |  |
| **Socioeconomic Status** |  |  |  |  |  | 0.41  (0.23, 0.71) | -3.26  (-10.23, 3.57) |
| Less poor  (Upper 2 quintiles) | 883/17,510  (5.0) | 1 | 37/53  (69.8) | 13.11  (8.98, 19.15) | 13.11  (8.98, 19.15) |  |  |
| Poorer  (Lower 3 quintiles) | 1,909/11,442  (17) | 2.04  (1.80, 2.30) | 31/39  (79) | 10.89  (7.18, 16.52) | 5.34  (3.55, 8.04) |  |  |
| **No HoH Education** |  |  |  |  |  | 0.28  (0.14, 0.54) | -5.98  (-11.70, 0.98) |
| Any education | 1,343/22,082  (6.1) | 1 | 53/69  (76.8) | 12.50  (9.13, 17.13) | 12.50  (9.13, 17.13) |  |  |
| No education | 1,449/6,870  (21) | 2.23  (2.02, 2.45) | 15/23  (65) | 7.75  (4.33, 13.90) | 3.48  (1.94, 6.24) |  |  |
| **Site** |  |  |  |  |  | 0.26  (0.13, 0.50) | -4.03  (-11.12, 5.88) |
| Timiri | 1,179/20,679  (5.7) | 1 | 53/71  (74.6) | 13.15  (9.55, 18.11) | 13.15  (9.55, 18.11) |  |  |
| Jawadhu Hills | 1,613/8,273  (19) | 3.42  (3.13, 3.73) | 15/21  (71) | 11.54  (6.39, 20.86) | 3.38  (1.87, 6.10) |  |  |

*Adjusted for age category, gender, and site; PR = Prevalence Ratio; CI = Confidence Interval; FD = Functional Difficulty; EM = Effect Modification

Table A8: Modification of the effect of functional-difficulty domain 'Concentrating' on school non-enrolment by selected sociodemographic factors among N=29,044 children in Tamil Nadu, India

(children with other functional limitations have been excluded).

|  | **No  Functional Difficulty** | | **Any  Functional Difficulty** | | **Effect of FD** | **EM Measures** | |
| --- | --- | --- | --- | --- | --- | --- | --- |
|  | **Non-enrolment  n/N**  **(%)** | **PR**  **(95% CI)** | **Non-enrolment  n/N**  **(%)** | **PR**  **(95% CI)** | **PR**  **(95% CI)** | **Multiplicative** | **Additive** |
| Overall  (crude) | 2,731/28,745  (10) | 1 | 62/84  (74) | 7.77  (6.77, 8.92) |  |  |  |
| Overall  (adjusted)* | 2,731/28,745  (10) | 1 | 62/84  (74) | 10.37  (8.14, 13.21) |  |  |  |
| **Age Category**  **(years)** |  |  |  |  |  | 0.20  (0.11, 0.36) | 2.64  (-19.07, 24.26) |
| 5-11 | 307/12,720  (2.4) | 1 | 26/40  (65.0) | 31.19  (19.56, 49.74) | 31.19  (19.56, 49.74) |  |  |
| 12-17 | 2,491/16,240  (15) | 6.37  (5.55, 7.31) | 36/44  (82) | 39.20  (26.21, 58.63) | 6.16  (4.19, 9.03) |  |  |
| **Gender** |  |  |  |  |  | 1.42  (0.79, 2.55) | 5.40  (-0.60, 12.37) |
| Female | 1,183/13,930  (8.5) | 1 | 28/41  (68.3) | 7.76  (5.03, 11.99) | 7.76  (5.03, 11.99) |  |  |
| Male | 1,615/15,030  (11) | 1.21  (1.11, 1.32) | 34/43  (79) | 13.37  (9.00, 19.87) | 11.02  (7.43, 16.35) |  |  |
| **Socioeconomic Status** |  |  |  |  |  | 0.45  (0.25, 0.82) | -2.07  (-9.83, 6.58) |
| Less poor  (Upper 2 quintiles) | 882/17,511  (5.0) | 1 | 38/52  (73.1) | 13.84  (9.53, 20.10) | 13.84  (9.53, 20.10) |  |  |
| Poorer  (Lower 3 quintiles) | 1,916/11,449  (17) | 2.05  (1.81, 2.31) | 24/32  (75) | 12.81  (8.02, 20.49) | 6.27  (3.94, 9.96) |  |  |
| **No HoH Education** |  |  |  |  |  | 0.27  (0.14, 0.53) | -6.90  (-13.67, 0.97) |
| Any education | 1,349/22,090  (6.1) | 1 | 47/61  (77.0) | 14.31  (10.26, 19.97) | 14.31  (10.26, 19.97) |  |  |
| No education | 1,449/6,870  (21) | 2.21  (2.00, 2.43) | 15/23  (65) | 8.62  (4.81, 15.44) | 3.91  (2.18, 6.99) |  |  |
| **Site** |  |  |  |  |  | 0.29  (0.14, 0.61) | -2.37  (-10.76, 10.87) |
| Timiri | 1,182/20,683  (5.7) | 1 | 50/67  (74.6) | 13.61  (9.80, 18.90) | 13.61  (9.80, 18.90) |  |  |
| Jawadhu Hills | 1,616/8,277  (20) | 3.41  (3.13, 3.73) | 12/17  (71) | 13.65  (7.06, 26.42) | 4.00  (2.07, 7.73) |  |  |

*Adjusted for age category, gender, and site; PR = Prevalence Ratio; CI = Confidence Interval; FD = Functional Difficulty; EM = Effect Modification

Table A9: Modification of the effect of functional-difficulty domain 'Accepting Change' on school non-enrolment by selected sociodemographic factors among N=29,044 children in Tamil Nadu, India

(children with other functional limitations have been excluded).

|  | **No  Functional Difficulty** | | **Any  Functional Difficulty** | | **Effect of FD** | **EM Measures** | |
| --- | --- | --- | --- | --- | --- | --- | --- |
|  | **Non-enrolment  n/N**  **(%)** | **PR**  **(95% CI)** | **Non-enrolment  n/N**  **(%)** | **PR**  **(95% CI)** | **PR**  **(95% CI)** | **Multiplicative** | **Additive** |
| Overall  (crude) | 2,731/28,745  (10) | 1 | 57/73  (78) | 8.22  (7.23, 9.34) |  |  |  |
| Overall  (adjusted)* | 2,731/28,745  (10) | 1 | 57/73  (78) | 9.08  (6.79, 12.15) |  |  |  |
| **Age Category**  **(years)** |  |  |  |  |  | 0.23  (0.12, 0.43) | 6.48  (-15.13, 28.27) |
| 5-11 | 311/12,727  (2.4) | 1 | 22/33  (66.7) | 27.52  (16.57, 45.68) | 27.52  (16.57, 45.68) |  |  |
| 12-17 | 2,492/16,244  (15) | 6.29  (5.48, 7.22) | 35/40  (88) | 39.28  (26.08, 59.17) | 6.25  (4.22, 9.24) |  |  |
| **Gender** |  |  |  |  |  | 1.10  (0.59, 2.04) | 2.62  (-3.56, 9.06) |
| Female | 1,186/13,938  (8.5) | 1 | 25/33  (75.8) | 8.44  (5.32, 13.40) | 8.44  (5.32, 13.40) |  |  |
| Male | 1,617/15,033  (11) | 1.21  (1.11, 1.32) | 32/40  (80) | 11.27  (7.48, 16.98) | 9.29  (6.17, 13.98) |  |  |
| **Socioeconomic Status** |  |  |  |  |  | 0.42  (0.22, 0.77) | -3.20  (-11.39, 5.26) |
| Less poor  (Upper 2 quintiles) | 887/17,521  (5.1) | 1 | 33/42  (78.6) | 14.12  (9.45, 21.10) | 14.12  (9.45, 21.10) |  |  |
| Poorer  (Lower 3 quintiles) | 1,916/11,450  (17) | 2.04  (1.80, 2.30) | 24/31  (77) | 11.95  (7.45, 19.18) | 5.87  (3.68, 9.35) |  |  |
| **No HoH Education** |  |  |  |  |  | 0.29  (0.14, 0.62) | -5.98  (-12.80, 3.25) |
| Any education | 1,350/22,095  (6.1) | 1 | 46/56  (82.1) | 13.35  (9.52, 18.74) | 13.35  (9.52, 18.74) |  |  |
| No education | 1,453/6,876  (21) | 2.22  (2.01, 2.45) | 11/17  (65) | 8.59  (4.33, 17.05) | 3.87  (1.95, 7.68) |  |  |
| **Site** |  |  |  |  |  | 0.37  (0.18, 0.74) | 0.81  (-8.26, 14.40) |
| Timiri | 1,190/20,696  (5.7) | 1 | 42/54  (77.8) | 12.93  (9.03, 18.52) | 12.93  (9.03, 18.52) |  |  |
| Jawadhu Hills | 1,613/8,275  (19) | 3.39  (3.10, 3.70) | 15/19  (79) | 16.13  (8.90, 29.23) | 4.76  (2.63, 8.62) |  |  |

*Adjusted for age category, gender, and site; PR = Prevalence Ratio; CI = Confidence Interval; FD = Functional Difficulty; EM = Effect Modification

Table A10: Modification of the effect of functional-difficulty domain 'Controlling Behaviour' on school non-enrolment by selected sociodemographic factors among N=29,044 children in Tamil Nadu, India

(children with other functional limitations have been excluded).

|  | **No  Functional Difficulty** | | **Any  Functional Difficulty** | | **Effect of FD** | **EM Measures** | |
| --- | --- | --- | --- | --- | --- | --- | --- |
|  | **Non-enrolment  n/N**  **(%)** | **PR**  **(95% CI)** | **Non-enrolment  n/N**  **(%)** | **PR**  **(95% CI)** | **PR**  **(95% CI)** | **Multiplicative** | **Additive** |
| Overall  (crude) | 2,731/28,745  (10) | 1 | 57/85  (67) | 7.06  (6.04, 8.24) |  |  |  |
| Overall  (adjusted)* | 2,731/28,745  (10) | 1 | 57/85  (67) | 8.96  (6.84, 11.74) |  |  |  |
| **Age Category**  **(years)** |  |  |  |  |  | 0.25  (0.13, 0.48) | 8.48  (-11.53, 29.04) |
| 5-11 | 312/12,721  (2.5) | 1 | 21/39  (53.8) | 24.36  (14.51, 40.88) | 24.36  (14.51, 40.88) |  |  |
| 12-17 | 2,491/16,238  (15) | 6.27  (5.46, 7.19) | 36/46  (78) | 38.11  (25.43, 57.10) | 6.08  (4.14, 8.94) |  |  |
| **Gender** |  |  |  |  |  | 1.13  (0.61, 2.09) | 2.68  (-3.12, 8.80) |
| Female | 1,186/13,931  (8.5) | 1 | 25/40  (62.5) | 7.87  (4.96, 12.48) | 7.87  (4.96, 12.48) |  |  |
| Male | 1,617/15,028  (11) | 1.21  (1.11, 1.32) | 32/45  (71) | 10.76  (7.14, 16.21) | 8.87  (5.89, 13.35) |  |  |
| **Socioeconomic Status** |  |  |  |  |  | 0.45  (0.24, 0.85) | -1.95  (-9.22, 6.21) |
| Less poor  (Upper 2 quintiles) | 885/17,509  (5.1) | 1 | 35/54  (64.8) | 12.34  (8.36, 18.24) | 12.34  (8.36, 18.24) |  |  |
| Poorer  (Lower 3 quintiles) | 1,918/11,450  (17) | 2.04  (1.80, 2.30) | 22/31  (71) | 11.43  (6.99, 18.70) | 5.61  (3.45, 9.13) |  |  |
| **No HoH Education** |  |  |  |  |  | 0.31  (0.15, 0.64) | -4.99  (-11.35, 3.16) |
| Any education | 1,352/22,088  (6.1) | 1 | 44/63  (69.8) | 12.20  (8.63, 17.25) | 12.20  (8.63, 17.25) |  |  |
| No education | 1,451/6,871  (21) | 2.21  (2.00, 2.43) | 13/22  (59) | 8.42  (4.48, 15.81) | 3.82  (2.03, 7.17) |  |  |
| **Site** |  |  |  |  |  | 0.41  (0.20, 0.85) | 2.04  (-6.62, 16.29) |
| Timiri | 1,188/20,682  (5.7) | 1 | 44/68  (64.7) | 11.16  (7.86, 15.86) | 11.16  (7.86, 15.86) |  |  |
| Jawadhu Hills | 1,615/8,277  (20) | 3.39  (3.11, 3.70) | 13/17  (76) | 15.60  (8.24, 29.53) | 4.60  (2.43, 8.69) |  |  |

*Adjusted for age category, gender, and site; PR = Prevalence Ratio; CI = Confidence Interval; FD = Functional Difficulty; EM = Effect Modification

Table A11: Modification of the effect of functional-difficulty domain 'Making Friends' on school non-enrolment by selected sociodemographic factors among N=29,044 children in Tamil Nadu, India

(children with other functional limitations have been excluded).

|  | **No  Functional Difficulty** | | **Any  Functional Difficulty** | | **Effect of FD** | **EM Measures** | |
| --- | --- | --- | --- | --- | --- | --- | --- |
|  | **Non-enrolment  n/N**  **(%)** | **PR**  **(95% CI)** | **Non-enrolment  n/N**  **(%)** | **PR**  **(95% CI)** | **PR**  **(95% CI)** | **Multiplicative** | **Additive** |
| Overall  (crude) | 2,731/28,745  (10) | 1 | 58/83  (70) | 7.36  (6.32, 8.56) |  |  |  |
| Overall  (adjusted)* | 2,731/28,745  (10) | 1 | 58/83  (70) | 9.23  (7.27, 11.71) |  |  |  |
| **Age Category**  **(years)** |  |  |  |  |  | 0.27  (0.14, 0.52) | 9.88  (-9.62, 29.35) |
| 5-11 | 314/12,725  (2.5) | 1 | 19/35  (54.3) | 22.53  (13.09, 38.76) | 22.53  (13.09, 38.76) |  |  |
| 12-17 | 2,488/16,236  (15) | 6.22  (5.42, 7.14) | 39/48  (81) | 37.62  (25.47, 55.58) | 6.05  (4.17, 8.76) |  |  |
| **Gender** |  |  |  |  |  | 1.16  (0.63, 2.13) | 2.81  (-2.55, 8.83) |
| Female | 1,183/13,929  (8.5) | 1 | 28/42  (66.7) | 7.39  (4.77, 11.45) | 7.39  (4.77, 11.45) |  |  |
| Male | 1,619/15,032  (11) | 1.22  (1.12, 1.33) | 30/41  (73) | 10.42  (6.82, 15.91) | 8.56  (5.61, 13.06) |  |  |
| **Socioeconomic Status** |  |  |  |  |  | 0.56  (0.30, 1.05) | 0.67  (-6.40, 9.22) |
| Less poor  (Upper 2 quintiles) | 885/17,510  (5.1) | 1 | 35/53  (66.0) | 11.02  (7.45, 16.30) | 11.02  (7.45, 16.30) |  |  |
| Poorer  (Lower 3 quintiles) | 1,917/11,451  (17) | 2.05  (1.81, 2.31) | 23/30  (77) | 12.73  (7.85, 20.65) | 6.23  (3.86, 10.03) |  |  |
| **No HoH Education** |  |  |  |  |  | 0.30  (0.14, 0.62) | -5.30  (-11.34, 2.63) |
| Any education | 1,350/22,090  (6.1) | 1 | 46/61  (75.4) | 11.86  (8.45, 16.65) | 11.86  (8.45, 16.65) |  |  |
| No education | 1,452/6,871  (21) | 2.21  (2.01, 2.44) | 12/22  (55) | 7.78  (4.03, 14.99) | 3.51  (1.82, 6.77) |  |  |
| **Site** |  |  |  |  |  | 0.36  (0.18, 0.72) | -0.01  (-7.93, 12.17) |
| Timiri | 1,188/20,687  (5.7) | 1 | 44/63  (69.8) | 11.44  (8.05, 16.27) | 11.44  (8.05, 16.27) |  |  |
| Jawadhu Hills | 1,614/8,274  (20) | 3.39  (3.11, 3.70) | 14/20  (70) | 13.83  (7.47, 25.60) | 4.07  (2.20, 7.54) |  |  |

*Adjusted for age category, gender, and site; PR = Prevalence Ratio; CI = Confidence Interval; FD = Functional Difficulty; EM = Effect Modification

Table A12: Modification of the effect of functional-difficulty domain 'Anxiety' on school non-enrolment by selected sociodemographic factors among N=29,044 children in Tamil Nadu, India

(children with other functional limitations have been excluded).

|  | **No  Functional Difficulty** | | **Any  Functional Difficulty** | | **Effect of FD** | **EM Measures** | |
| --- | --- | --- | --- | --- | --- | --- | --- |
|  | **Non-enrolment  n/N**  **(%)** | **PR**  **(95% CI)** | **Non-enrolment  n/N**  **(%)** | **PR**  **(95% CI)** | **PR**  **(95% CI)** | **Multiplicative** | **Additive** |
| Overall  (crude) | 2,731/28,745  (10) | 1 | 31/42  (74) | 7.77  (6.48, 9.32) |  |  |  |
| Overall  (adjusted)* | 2,731/28,745  (10) | 1 | 31/42  (74) | 6.97  (4.74, 10.24) |  |  |  |
| **Age Category**  **(years)** |  |  |  |  |  | 0.16  (0.07, 0.39) | -4.85  (-32.65, 18.09) |
| 5-11 | 321/12,744  (2.5) | 1 | 12/16  (75.0) | 26.64  (13.53, 52.47) | 26.64  (13.53, 52.47) |  |  |
| 12-17 | 2,508/16,258  (15) | 6.14  (5.35, 7.03) | 19/26  (73) | 26.94  (15.63, 46.42) | 4.39  (2.58, 7.46) |  |  |
| **Gender** |  |  |  |  |  | 1.01  (0.44, 2.33) | 1.26  (-5.19, 7.89) |
| Female | 1,197/13,951  (8.6) | 1 | 14/20  (70.0) | 6.45  (3.48, 11.96) | 6.45  (3.48, 11.96) |  |  |
| Male | 1,632/15,051  (11) | 1.21  (1.11, 1.32) | 17/22  (77) | 7.92  (4.52, 13.89) | 6.53  (3.73, 11.43) |  |  |
| **Socioeconomic Status** |  |  |  |  |  | 0.23  (0.10, 0.54) | -8.67  (-18.87, 0.16) |
| Less poor  (Upper 2 quintiles) | 900/17,538  (5.1) | 1 | 20/25  (80.0) | 14.36  (8.58, 24.04) | 14.36  (8.58, 24.04) |  |  |
| Poorer  (Lower 3 quintiles) | 1,929/11,464  (17) | 2.03  (1.80, 2.30) | 11/17  (65) | 6.73  (3.35, 13.50) | 3.31  (1.66, 6.60) |  |  |
| **No HoH Education** |  |  |  |  |  | 0.28  (0.10, 0.79) | -5.19  (-12.33, 5.29) |
| Any education | 1,371/22,117  (6.2) | 1 | 25/34  (73.5) | 10.37  (6.56, 16.40) | 10.37  (6.56, 16.40) |  |  |
| No education | 1,458/6,885  (21) | 2.20  (2.00, 2.43) | 6/8  (75) | 6.39  (2.52, 16.19) | 2.90  (1.15, 7.34) |  |  |
| **Site** |  |  |  |  |  | 0.27  (0.11, 0.66) | -3.42  (-13.16, 9.29) |
| Timiri | 1,211/20,723  (5.8) | 1 | 21/27  (77.8) | 12.07  (7.27, 20.02) | 12.07  (7.27, 20.02) |  |  |
| Jawadhu Hills | 1,618/8,279  (20) | 3.34  (3.06, 3.65) | 10/15  (67) | 10.99  (5.30, 22.82) | 3.29  (1.59, 6.82) |  |  |

*Adjusted for age category, gender, and site; PR = Prevalence Ratio; CI = Confidence Interval; FD = Functional Difficulty; EM = Effect Modification

Table A13: Modification of the effect of functional-difficulty domain 'Depression' on school non-enrolment by selected sociodemographic factors among N=29,044 children in Tamil Nadu, India

(children with other functional limitations have been excluded).

|  | **No  Functional Difficulty** | | **Any  Functional Difficulty** | | **Effect of FD** | **EM Measures** | |
| --- | --- | --- | --- | --- | --- | --- | --- |
|  | **Non-enrolment  n/N**  **(%)** | **PR**  **(95% CI)** | **Non-enrolment  n/N**  **(%)** | **PR**  **(95% CI)** | **PR**  **(95% CI)** | **Multiplicative** | **Additive** |
| Overall  (crude) | 2,731/28,745  (10) | 1 | 22/29  (76) | 7.98  (6.52, 9.78) |  |  |  |
| Overall  (adjusted)* | 2,731/28,745  (10) | 1 | 22/29  (76) | 7.76  (4.98, 12.09) |  |  |  |
| **Age Category**  **(years)** |  |  |  |  |  | 0.11  (0.04, 0.30) | -17.00  (-59.66, 14.07) |
| 5-11 | 323/12,749  (2.5) | 1 | 10/11  (90.9) | 37.43  (17.85, 78.51) | 37.43  (17.85, 78.51) |  |  |
| 12-17 | 2,515/16,266  (15) | 6.12  (5.34, 7.01) | 12/18  (67) | 25.55  (12.97, 50.35) | 4.18  (2.14, 8.15) |  |  |
| **Gender** |  |  |  |  |  | 1.22  (0.45, 3.28) | 2.90  (-4.95, 13.62) |
| Female | 1,199/13,956  (8.6) | 1 | 12/15  (80.0) | 6.44  (3.30, 12.56) | 6.44  (3.30, 12.56) |  |  |
| Male | 1,639/15,059  (11) | 1.22  (1.11, 1.33) | 10/14  (71) | 9.55  (4.60, 19.84) | 7.85  (3.78, 16.30) |  |  |
| **Socioeconomic Status** |  |  |  |  |  | 0.24  (0.08, 0.68) | -8.48  (-20.88, 3.14) |
| Less poor  (Upper 2 quintiles) | 905/17,545  (5.2) | 1 | 15/18  (83.3) | 14.49  (8.00, 26.23) | 14.49  (8.00, 26.23) |  |  |
| Poorer  (Lower 3 quintiles) | 1,933/11,470  (17) | 2.02  (1.79, 2.29) | 7/11  (64) | 7.02  (2.94, 16.76) | 3.47  (1.46, 8.24) |  |  |
| **No HoH Education** |  |  |  |  |  | 0.20  (0.06, 0.70) | -8.07  (-17.47, 4.21) |
| Any education | 1,378/22,129  (6.2) | 1 | 18/22  (81.8) | 12.16  (7.09, 20.85) | 12.16  (7.09, 20.85) |  |  |
| No education | 1,460/6,886  (21) | 2.19  (1.99, 2.42) | 4/7  (57) | 5.28  (1.69, 16.49) | 2.41  (0.77, 7.52) |  |  |
| **Site** |  |  |  |  |  | 0.32  (0.11, 0.91) | -1.74  (-13.82, 16.36) |
| Timiri | 1,217/20,730  (5.9) | 1 | 15/20  (75.0) | 11.85  (6.52, 21.56) | 11.85  (6.52, 21.56) |  |  |
| Jawadhu Hills | 1,621/8,285  (20) | 3.33  (3.05, 3.64) | 7/9  (78) | 12.44  (5.20, 29.79) | 3.74  (1.56, 8.94) |  |  |

*Adjusted for age category, gender, and site; PR = Prevalence Ratio; CI = Confidence Interval; FD = Functional Difficulty; EM = Effect Modification
